# Pharmacological inhibition of LSD1 for the treatment of MLL-rearranged leukemia

**DOI:** 10.1186/s13045-016-0252-7

**Published:** 2016-03-12

**Authors:** Zizhen Feng, Yuan Yao, Chao Zhou, Fengju Chen, Fangrui Wu, Liping Wei, Wei Liu, Shuo Dong, Michele Redell, Qianxing Mo, Yongcheng Song

**Affiliations:** Department of Pharmacology, Baylor College of Medicine, 1 Baylor Plaza, Houston, TX 77030 USA; Dan L. Duncan Cancer Center, Baylor College of Medicine, 1 Baylor Plaza, Houston, TX 77030 USA; Department of Pediatrics, Baylor College of Medicine, 1 Baylor Plaza, Houston, TX 77030 USA; Texas Children’s Cancer and Hematology Centers, 1102 Bates Street, Houston, TX 77030 USA; Department of Medicine, Baylor College of Medicine, 1 Baylor Plaza, Houston, TX 77030 USA

**Keywords:** MLL-rearranged leukemia, Lysine-specific demethylase 1, Enzyme inhibitor, Drug discovery, Leukemia therapeutics

## Abstract

**Background:**

Mixed lineage leukemia (MLL) gene translocations are found in ~75 % infant and 10 % adult acute leukemia, showing a poor prognosis. Lysine-specific demethylase 1 (LSD1) has recently been implicated to be a drug target for this subtype of leukemia. More studies using potent LSD1 inhibitors against MLL-rearranged leukemia are needed.

**Methods:**

LSD1 inhibitors were examined for their biochemical and biological activities against LSD1 and MLL-rearranged leukemia as well as other cancer cells.

**Results:**

Potent LSD1 inhibitors with biochemical IC_50_ values of 9.8–77 nM were found to strongly inhibit proliferation of MLL-rearranged leukemia cells with EC_50_ of 10–320 nM, while these compounds are generally non-cytotoxic to several other tumor cells. LSD1 inhibition increased histone H3 lysine 4 (H3K4) methylation, downregulated expression of several leukemia-relevant genes, induced apoptosis and differentiation, and inhibited self-renewal of stem-like leukemia cells. Moreover, LSD1 inhibitors worked synergistically with inhibition of DOT1L, a histone H3 lysine 79 (H3K79) methyltransferase, against MLL-rearranged leukemia. The most potent LSD1 inhibitor showed significant in vivo activity in a systemic mouse model of MLL-rearranged leukemia without overt toxicities. Mechanistically, LSD1 inhibitors caused significant upregulation of several pathways that promote hematopoietic differentiation and apoptosis.

**Conclusions:**

LSD1 is a drug target for MLL-rearranged leukemia, and LSD1 inhibitors are potential therapeutics for the malignancy.

**Electronic supplementary material:**

The online version of this article (doi:10.1186/s13045-016-0252-7) contains supplementary material, which is available to authorized users.

## Background

Acute leukemia, including acute lymphoblastic leukemia (ALL) and acute myeloid leukemia (AML), afflicts people of all ages, and it is the most common cancer affecting children under the age of 15. Although certain subtypes of leukemia, e.g., childhood ALL, have achieved high cure rates, the 5-year survival rates for the majority of acute leukemia (mostly AML) patients are still low [1]. With a few exceptions, current treatments are conventional chemotherapeutics, which non-selectively kill all rapidly proliferating cells including normal stem/progenitor cells in the bone marrow and other organs (e.g., intestines). This causes severe toxicities and side effects, usually limiting the efficacy of these drugs. Biomarkers, such as mixed lineage leukemia (MLL) gene translocation and MEF2C [2], are frequently used to classify molecular subtypes of AML, predict prognosis, and determine therapeutic regimes. Acute leukemia carrying an MLL gene translocation accounts for the majority (~75 %) of leukemia in infants as well as ~10 % in children and adults. This subtype of leukemia shows a poor prognosis, with 5-year event-free survival being only ~40 % [3–5]. Intensified chemotherapy causes increased toxicity to patients without a significant improvement of survival. There is therefore a pressing need to find new therapeutics.

MLL is a large protein (3969 amino acid residues) with multiple domains (Additional file 1: Figure S1A and B). Its C-terminal SET domain, a homolog of *Drosophila* trithorax, is a histone H3 lysine 4 (H3K4) methyltransferase. The N-terminal AT hook domain recognizes the promoters or enhancers of certain genes and directs the methylation loci for the SET domain [6]. Studies show that methylated H3K4 (H3K4me1, 2, or 3) is associated with active transcription of many genes including Hox genes important for hematopoiesis [7, 8]. However, overexpression of certain Hox genes, such as HoxA9, leads to leukemogenesis [9]. Cellular H3K4 methylation is therefore tightly regulated. For example, MLL is assembled as a member of a large protein complex (with ≥29 proteins) containing lysine-specific demethylase 1 (LSD1, also known as KDM1a) [10], which can demethylate H3K4me1 and 2 (but not H3K4me3) and plays an opposite role in keeping a balanced H3K4 methylation status [11] (Additional file 1: Figure S1B). In MLL-rearranged leukemia, the onco-MLL loses the SET domain and is fused with one of the >70 documented genes (Additional file 1: Figure S1C), with AF4, AF10, AF9, and its homolog ENL being predominant (>70 %) [6, 12]. The mechanism for MLL leukemia has been well studied [9, 13, 14]. These MLL fusion partners are able to recruit DOT1L, a histone H3 lysine 79 (H3K79) methyltransferase (Additional file 1: Figure S1D). This leads to aberrant H3K79 methylation at MLL target gene loci, causing dysregulated gene expression (e.g., overexpression of HoxA9 and Meis1) and eventually initiation of the leukemia. Indeed, potent small molecule inhibitors of DOT1L, developed by us [15–17] and others [18–21], have been found to have selective activity against MLL leukemia.

LSD1 is a flavin adenine dinucleotide (FAD)-dependent monoamine oxidase (MAO), and its mechanism of catalysis is illustrated in Additional file 1: Figure S2 [11, 22]. The methyl group in H3K4me1 or 2 is removed by FAD-mediated oxidation, after which FAD is regenerated by oxidation with O_2_ to complete a catalytic cycle. The biological function of LSD1 is crucial, as LSD1 knockout in mice is embryonic lethal and conditional knockout blocked hematopoiesis [23]. Overexpression of LSD1 was found in several types of cancers (e.g., prostate and breast), suggesting that LSD1 might be a drug target for intervention [24–26]. Recently, LSD1 was reported to be required for leukemia stem cells (LSC) with MLL-AF9 fusion oncogene [27]. Using cyclopropylamine-based LSD1 inhibitors also showed in vitro and in vivo activity against MLL-AF9 leukemia. However, the compounds in the study exhibited severe toxicity, with many of the experimental mice dying of severe anemia/thrombocytopenia. More studies are therefore needed to show that this chemotype of LSD1 inhibitors can be safely used in the clinic [28, 29].

Here, we synthesized a series of cyclopropylamine-based LSD1 inhibitors and found that these compounds possess potent and selective activity against MLL-rearranged leukemia, with their antileukemia activities correlated with LSD1 inhibitory activity. In addition, we show that one compound exhibited significant in vivo activity in a mouse model of MLL leukemia without obvious toxicities, showing that potent LSD1 inhibitors are potentially useful therapeutics for this subtype of acute leukemia. Molecular and cell biology studies were performed to characterize these compounds in MLL-rearranged leukemia as well as possible mechanism(s) of action.

## Results

### LSD1 inhibitors exhibited potent antileukemia activity

**Fig. 1:**
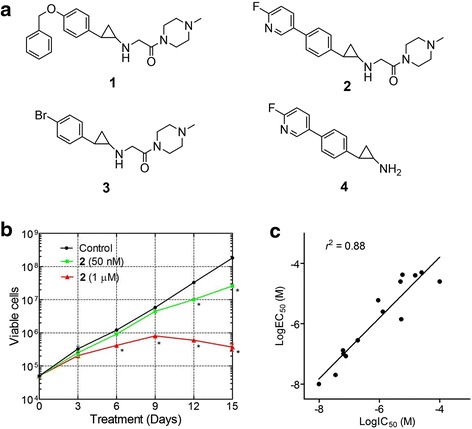
LSD1 inhibitors blocked proliferation of MV4-11 leukemia cells. **a** Structures of compounds **1**–**4. b** Growth curves for MV4-11 cells with or without treatment with compound **2** (* *p* < 0.01 with respect to the control). **c** Good correlation between enzyme IC_50_ values of LSD1 inhibitors and their antiproliferative activities, showing a *R* ^2^ of 0.88

A number of several chemotypes of LSD1 inhibitors have been reported [30–37], among which cyclopropylamine-containing compounds exhibited low nM IC_50_ values against the enzyme. However, these compounds have not been evaluated for their activity against leukemia cells. We synthesized compounds **1–3** (Fig. 1a) and tested their biochemical inhibition against recombinant human LSD1. Choosing these three compounds was based on their reported low nanometer inhibitory activity against LSD1 [30]. The LSD1 inhibition assay was performed with the reaction rate (i.e., amount of the product H_2_O_2_, Additional file 1: Figure S2) being quantitatively determined by adding horseradish peroxidase (HRP) and a HRP fluorescence substrate Amplex red. Thus, compound **1** with a flexible 4-benzyloxy group was found to be an extremely potent inhibitor with an IC_50_ value of 9.8 nM (Table 1), which almost quantitatively deactivates LSD1 (~30 nM in the assay). Compound 2 having a rigid fluoropyridine substituent is also a potent inhibitor with an IC_50_ of 77 nM. Compound 3 with a less bulky 4-Br group exhibited 2× more activity (IC_50_ = 35 nM) than 2. These results are comparable to those reported previously [30].

**Table 1:** LSD1 inhibitory (IC_50_, μM) and antiproliferative (15-day treatment EC_50_, μM) activities for compounds **1**–**4**

	LSD1 IC_50_	MLL-rearranged leukemia		Non-MLL leukemia cells		Breast	Prostate
		MV4-11	Molm-13	NB4	U937	MCF-7	LNCaP
1	0.0098	0.010	0.096	19.4	18.9	1.6	3.7
2	0.077	0.084	0.32	18.9	34.6	6.6	10.0
3	0.035	0.020	0.18	>50	>50	13.5	29.9
4	5.3	1.4	12	>50	17.5	>50	>50

Next, these three compounds were tested for their cellular activity against proliferation of MV4-11 leukemia cells bearing MLL-AF4 fusion oncogene. As representatively shown in Fig. 1b, unlike traditional chemotherapeutics that exhibit cytotoxicity within 2 days, the LSD1 inhibitors did not significantly affect proliferation of MV4-11 cells in a shorter period of time (e.g., 3 days), depending on their concentrations. However, upon incubation for ≥10 days, these compounds showed potent activity against MV4-11 cells with EC_50_ values for compounds **1–3** being 10, 84, and 20 nM, respectively (Table 1). The slow antiproliferative action suggests that these LSD1 inhibitors do not have general cytotoxicity, e.g., inhibition of DNA/RNA/protein biosynthesis that affects all rapidly proliferating cells. Rather, similar to DOT1L inhibitors [16, 18], a series of cellular events are required for LSD1 inhibitors to inhibit cell growth, which could include blocked H3K4 demethylation, downregulation of leukemia-relevant genes, and depletion of downstream effector proteins.

### Antileukemia activity correlated with LSD1 inhibitory activity

Given the potent activity of compounds **1–3**, more analogous compounds were investigated. While detailed structure activity relationships of these compounds will be described in a separate publication, we show the antileukemia data of several representative compounds, including compound **4** (Fig. 1a), which is a fragment of compound **2** without the second substituent at the amine-N atom. Compound **4** exhibited a good inhibitory activity against LSD1 with an IC_50_ value of 5.3 μM (Table 1), but it is ~70-fold less potent than compound **2**, showing the that the second substituent of **2** plays an important role in LSD1 inhibition. In addition, compound **4** was also found to have a significantly reduced antiproliferative activity (EC_50_, 1.4 μM) against MV4-11 leukemia cells.

Fourteen compounds (including compounds **1**–**4**) with a broad range of LSD1 inhibitory activities with IC_50_ values of 9.8 nM to >100 μM were selected and tested against proliferation of MV4-11 leukemia cells. The structures and biochemical IC_50_ values of compounds **5**–**14**, as well as their antiproliferative EC_50_ values, are summarized in Additional file 1: Table S1. Overall, as shown in Fig. 1c, LSD1 inhibition (enzyme IC_50_ values) for these 14 compounds was found to be well correlated with their antiproliferation activity (EC_50_ values), with the *R*^2^ value of 0.88. These results suggest that LSD1 could be the cellular target responsible for the cell growth inhibition.

### Potent and selective activity against MLL-rearranged leukemia

We next examined the spectrum of antitumor activity for compounds **1**–**4**. These compounds were incubated with a panel of six human leukemia and solid tumor cell lines, including two MLL-rearranged leukemia cells MV4-11 and Molm-13 containing fusion oncogenes MLL-AF4 and -AF9, respectively. NB4 and U937 leukemia cells do not have an MLL translocation. Also included in the studies are breast cancer MCF-7 and prostate cancer LNCaP cells. Similar to their activities against MV4-11, compounds **1**–**4** had no or negligible activity against proliferation of all other tumor cells in a 3-day treatment. However, as summarized in Table 1, potent LSD1 inhibitors **1**–**3** showed particularly high activities against MLL-rearranged leukemia cells MV4-11 and Molm-13 with EC_50_ values of 10–320 nM. The cell activities seem to be in line with the LSD1 inhibitory activities, with compound **1** being the most potent (EC_50_ = 10 and 96 nM against MV4-11 and Molm-13). Moreover, less potent LSD1 inhibitor **4** was found to possess considerably reduced activities against the two MLL-rearranged leukemia cells (EC_50_ = 1.4 and 12 μM).

NB4 and U937 leukemia cells without an MLL translocation exhibited low susceptibility to the treatment with compounds **1**–**3**. The 15-day treatment EC_50_ values for compounds **1**–**3** are ≥19 μM, showing that these compounds have high selectivity (>108-fold) against MLL-rearranged leukemia cells. LSD1 has been reported to be overexpressed in breast and prostate cancers [24, 25]. The most potent LSD1 inhibitor **1** showed good antiproliferative activity against MCF-7 (breast) and LNCaP (prostate) cancer cells with 15-day EC_50_ values of 1.6 and 3.7 μM, respectively. Compound **2** had moderate activity (15-day EC_50_, 6.6 and 10 μM) against these two cells, while compound **3** did not significantly affect MCF-7 and LNCaP cells (15-day EC_50_ = 13.5 and 29.9 μM). These results show that MLL-rearranged MV4-11 and Molm-13 leukemia cells are far more sensitive to LSD1 inhibition.

### Activity against MAO-A/-B and selectivity for LSD1

The cyclopropylamine LSD1 inhibitors were generally derived from MAO-A/-B inhibitor tranylcypromine, an antidepression drug. Because MAO-A and -B play important roles in the degradation of neurotransmitters (e.g., serotonin and dopamine) in the central nervous system, selective inhibition for LSD1 is highly desirable. We tested compounds **1**–**4** for their biochemical activity against human MAO-A and -B, and the results are summarized in Table 2. Compounds **1** and **2** exhibited only weak activity (IC_50_, 17.5–480 μM) against MAO-A and -B, showing excellent selectivity for LSD1 inhibition of >1500-folds. Compound **3** had moderate inhibitory activity (IC_50_, 7.3 and 16.3 μM) against these two MAO enzymes and it also showed >200× selectivity for LSD1. However, compound **4** without the second amine-N substituent exhibited more potent inhibitory activity against MAO-A and -B (IC_50_, 0.42 and 3.1 μM, respectively). These results indicate that compounds **1** and **2** are highly potent and selective LSD1 inhibitors.

**Table 2:** Inhibitory activity (IC_50_, μM) against MAO-A and -B for compounds **1**–**4** and their selectivity indices for LSD1

	LSD1	MAO-A	MAO-B	Selectivity index
1	0.0098	17.5	34.2	>1700
2	0.077	120	480	>1500
3	0.035	7.3	16.3	>208
4	5.3	0.42	3.1	<0.58

### Cell activities of LSD1 inhibitors

**Fig. 2:**
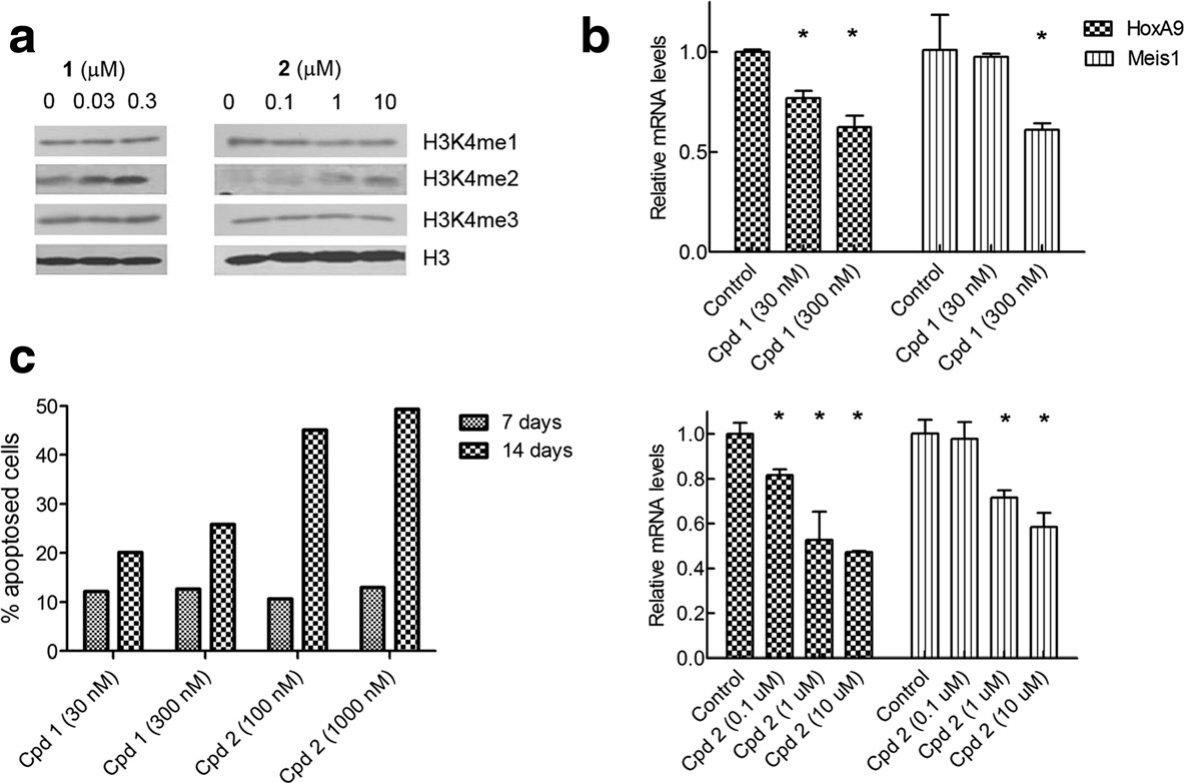
Dose-dependent biological activities of compounds **1** and **2** in MV4-11 cells. **a 1** and **2** caused increased levels of H3K4me2, while these compounds seem not to affect H3K4me1 and me3 significantly. **b 1** (*upper*) and **2** (*lower panel*) reduced gene expression of HoxA9 and Meis1 whose overexpression is characteristic to MLL-rearranged leukemia (* *p* < 0.05 with respect to the control). **c 1** and **2** induced considerable amounts of apoptosis in 7 or 14 days

Compounds **1** and **2** were selected for further biological activity testing. MV4-11 cells were treated with these two compounds for 3 days. Western blot experiments showed that both compounds **1** and **2** can increase cellular levels of H3K4me2 in a dose-dependent manner (Fig. 2a), indicating that these compounds are cell membrane permeable and inhibit LSD1 in MV4-11 cells. In addition, these compounds did not seem to consistently affect the global levels of H3K4me1 and H3K4me3. Other researchers observed the similar results using a different LSD1 inhibitor.

Next, we examined whether LSD1 inhibition can block the expression of HoxA9 and Meis1, whose overexpression has been observed in MLL-rearranged leukemia [9, 18, 20]. As shown in Fig. 2b, treatment with compounds **1** and **2** can significantly decrease the expression of both HoxA9 and Meis1 in a dose-dependent manner.

Third, compounds **1** and **2** were found to be able to induce significant apoptosis of MV4-11 cells using fluorescence-activated cell sorting (FACS)-based Annexin-V assay. As shown in Fig. 2c and Additional file 1: Figures S3 and S4, compound **1** at 30 nM can induce 12 % apoptosis on day 7 and showed an increased potency of 20 % on day 14. At 300 nM, **1** exhibited more potent activity in inducing apoptosis of MV4-11, being 13 % on day 7 and 26 % on day 14. Similarly, compound **2** at 0.1 μM induced 11 and 13 % apoptosis of MV4-11 cells in 7 and 14 days (respectively). It promoted significantly more apoptosis (45 and 49 % for 7 and 14 days) at 1 μM. These results suggest that LSD1 inhibition led to an increased methylation at H3K4 and this epigenetic change suppressed the expression of leukemia relevant genes and induced apoptosis of MLL-rearranged leukemia cells.

**Fig. 3:**
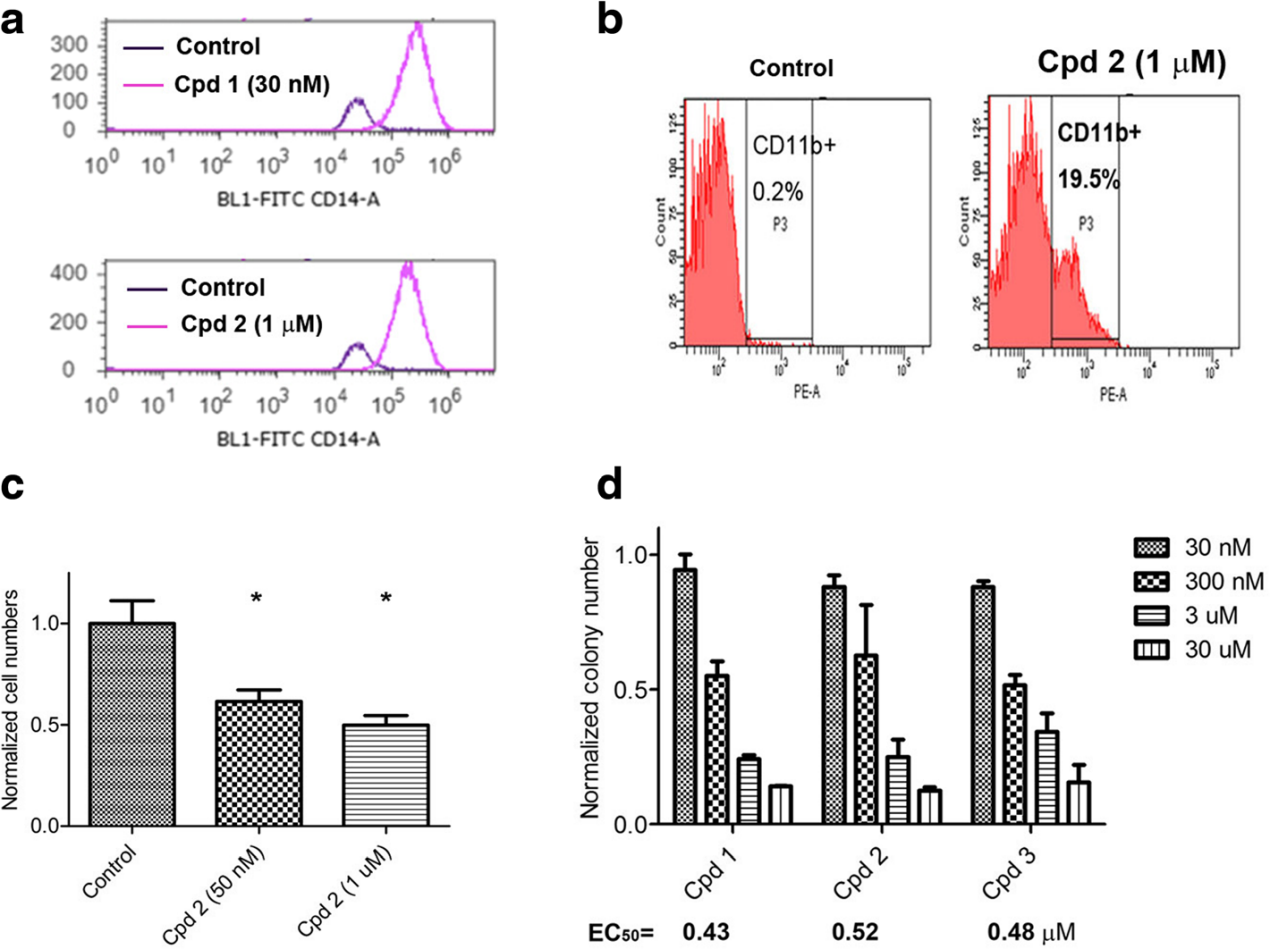
LSD1 inhibition promoted differentiation and inhibited cell migration and self-renewal. **a** Treatment of MV4-11 cells with compounds **1** and **2** for 12 days caused >90 % cell populations expressing high levels of CD14, a cell surface protein characteristic for macrophages/monocytes. **b** Treatment of MV4-11 cells with **2** led to significantly more cells expressing high levels of CD11b, a cell surface protein for macrophages/monocytes. **c** Treatment with **2** significantly reduced numbers of MV4-11 cells that can migrate through a membrane with 8-μm pores (* *p* < 0.05 with respect to the control). **d** Treatment of human primary leukemia cells (from an MLL-rearranged leukemia patient) with LSD1 inhibitors **1**–**3** potently inhibited the colony-forming ability with EC_50_ values of 0.43, 0.52, and 0.48 μM, respectively

Studies have shown that there are a small proportion of stem-like cancer cells (also known as cancer stem cells) within the bulk of cancer, which possess certain traits of normal stem cells, e.g., self-renewal and ability to differentiate [38, 39]. Importantly, only these stem-like cancer cells can form new tumors when transplanted into a new host, while the other non-stem cancer cells fail to do so. These stem-like cancer cells are more drug resistant and believed to be responsible for tumor metastasis and relapse. We found that LSD1 inhibition can induce differentiation of MV4-11 stem-like leukemia cells and inhibit cell migration. CD14 and CD11b are two cell surface proteins characteristic to differentiated macrophages/monocytes [18]. As shown in Fig. 3a, treatment with compounds **1** and **2** for 12 days caused significantly increased cells expressing high levels of CD14. Similarly, incubation with **2** led to considerably more CD11b^+^ cells (Fig. 3b). These results indicate that LSD1 inhibition can induce differentiation of stem-like leukemia cells to become more matured macrophage-like cells. In addition, treatment with compound **2** for 4 days led to reduced ability for MV4-11 cells to migrate through a membrane with 8-μm pores (Fig. 3c), showing that LSD1 inhibition could have the potential to block tumor cell migration.

### LSD1 inhibition reduced colony-forming ability of human primary MLL leukemia cells

Colony-forming assay using non-serum culture media represents a useful method to assess self-renewal capacity of stem-like cancer cells [27, 40]. The number of cell colonies formed in the assay reflects the proportion of stem-like cells in a given number of cancer cells. To make the assessment more clinically relevant, we determined the ability of compounds **1**–**3** to inhibit the colony-forming ability of human primary leukemia cells from an AML patient with MLL-ENL fusion oncogene; 10^4^ primary leukemia cells were plated in Methocult H4434 methylcellulose medium containing increasing concentrations of compounds **1**–**3** and colonies in each culture dish were counted after 14 days. As shown in Fig. 3d, potent LSD1 inhibitors **1**–**3** were able to largely reduce the numbers of cell colonies with EC_50_ values of 0.43, 0.52, and 0.48 μM, respectively, showing that LSD1 inhibition can impair the leukemia stem-like cells in the clinical sample.

### Synergy when combined with DOT1L inhibition

Previous biological studies have shown that H3K79 methyltransferase DOT1L is a drug target for MLL-rearranged leukemia [9, 16, 18]. Medicinal chemistry by us and others has identified several highly potent DOT1L inhibitors [15–21]. SYC-522 (Additional file 1: Figure S5) is a potent and specific inhibitor of DOT1L with K_i_ of 0.5 nM [16]. In our previous work, it can inhibit the H3K79 methylation in MV4-11 cells with an IC_50_ of ~200 nM [17]. SYC-522 also selectively blocked proliferation of MLL-rearranged leukemia cells, including MV4-11 and Molm-13 with the EC_50_ values of ~4 and 7 μM [16].

**Fig. 4:**
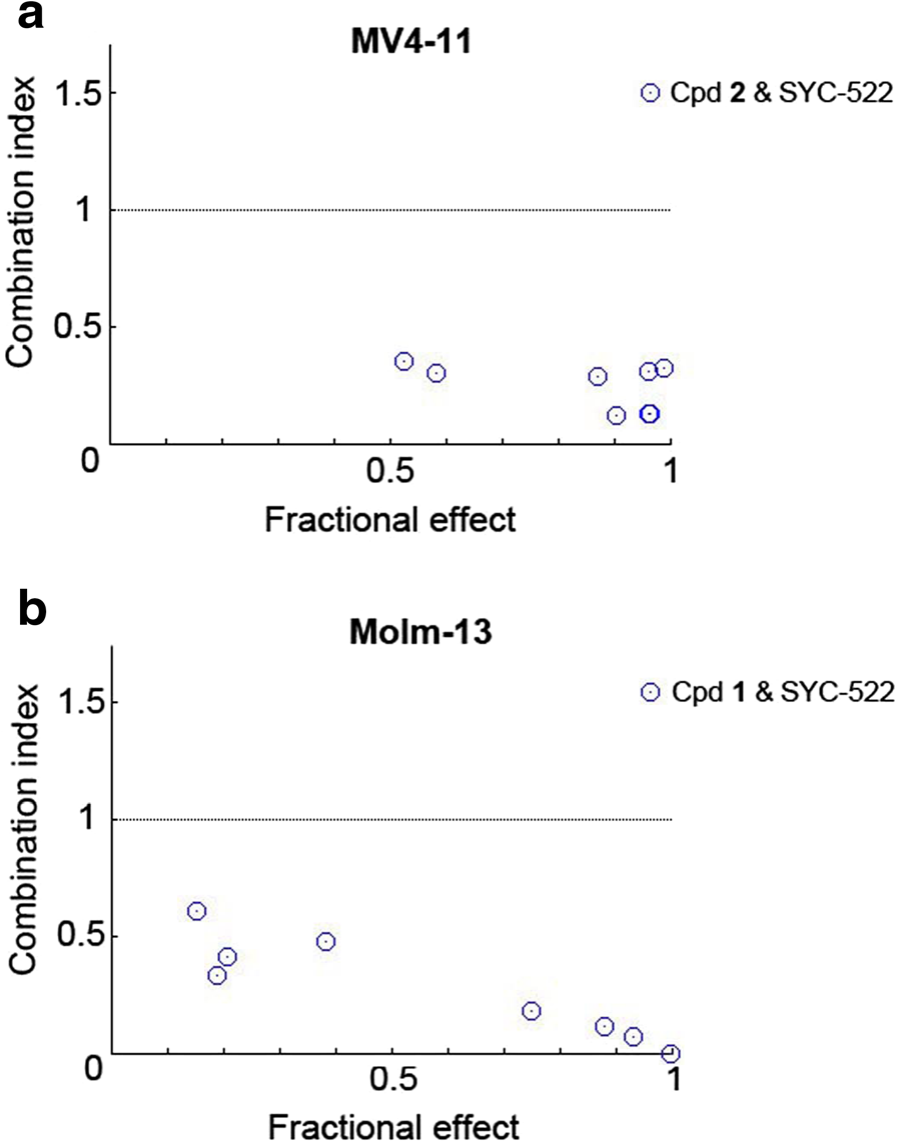
Combination of LSD1 and DOT1L inhibition caused synergistic effects against proliferation of MLL-rearranged leukemia cells. **a** Combination treatment of MV4-11 cells with compounds **2** and SYC-522 exhibited combination index (CI) values of 0.13–0.36. **b** Combination treatment of Molm-13 cells with compounds **1** and SYC-522 showed combination index (CI) values of 0.01–0.61. CI values <1 indicate synergism, while those =1 and >1 show additive effect and antagonism, respectively

We investigated the combination of LSD1 and DOT1L inhibition in MV4-11 and Molm-13 cells. These cells were treated with a matrix of increasing concentrations (from 0 to 0.1×, 0.33×, 1×, 3×, and 10 × EC_50_ for each individual compound) of compound **1** (or **2**) and SYC-522 for 15 days. Cell viability in each well was determined and data were analyzed by the program CompuSyn, which calculates the combination index (CI) for each drug combination [41]. The combinations with CI <1 indicate synergism for the two drugs, while those with CI = 1 show additive effect and those with CI >1 represent antagonism. As shown in Fig. 4a, combinations of compounds **2** and SYC-522 exhibited strong synergy in inhibiting proliferation of MV4-11 cells, with the CI values ranging from 0.13 to 0.36. Similarly, combinations of compounds **1** and SYC-522 also achieved strong synergy against Molm-13 cells (Fig. 4b). These results suggest that combination inhibition of these two enzymes can achieve synergism against this subtype of cancer.

### Compound **1** exhibited significant in vivo antileukemia activity without overt toxicity

**Fig. 5:**
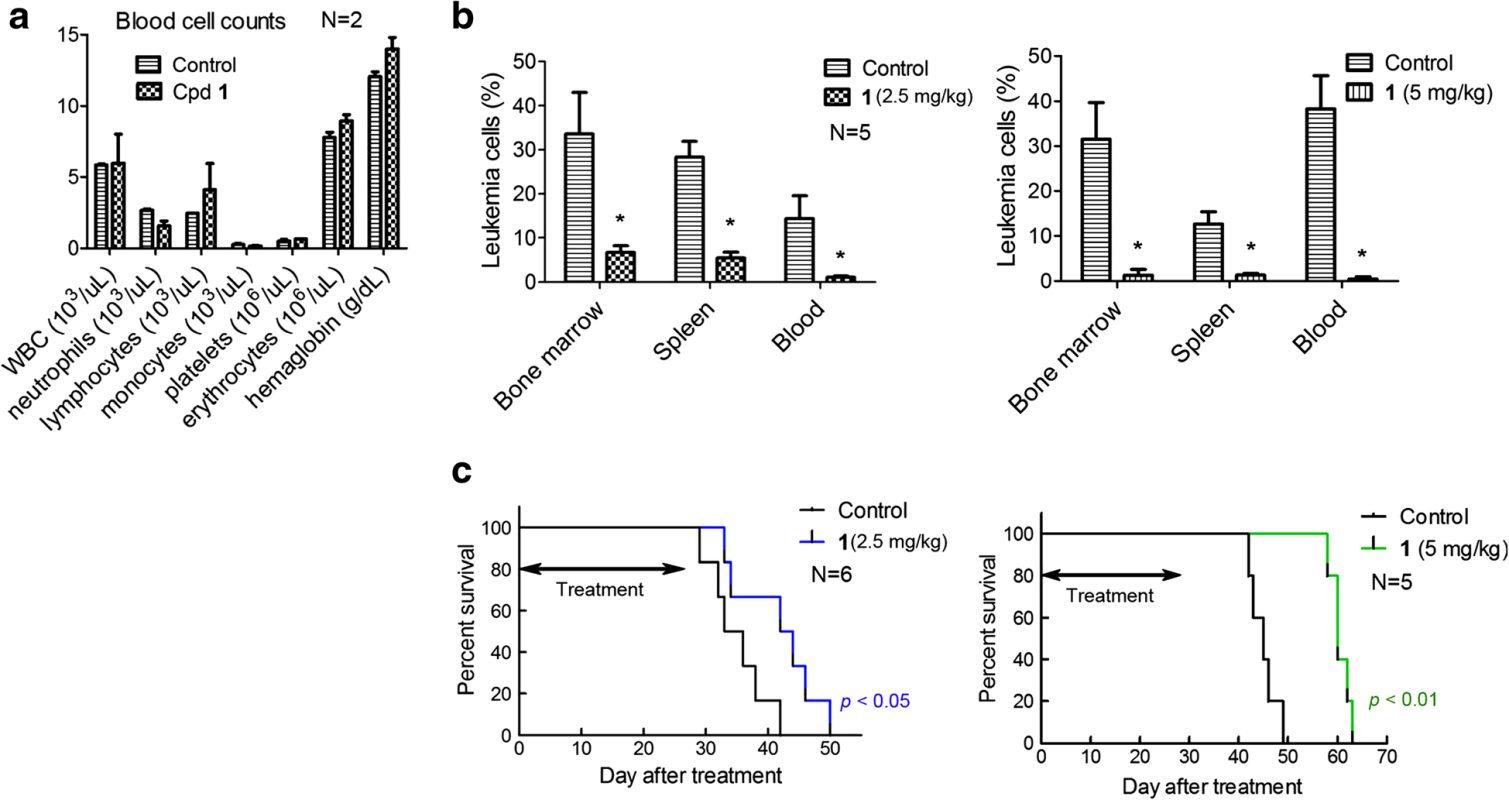
In vivo activities of compound **1** in a systemic mouse model of MV4-11 leukemia. **a** Treatment of NOD-SCID mice with **1** caused no significant changes in blood cell counts, suggesting no obvious toxicities. **b** Compound **1** (2.5 and 5 mg/kg/day for 28 days) significantly reduced MV4-11 leukemia cells in the bone marrow, spleen, and peripheral blood, with the higher dosage showing more pronounced antitumor effects (* *p* < 0.05 with respect to the control). **c** Treatment with **1** (2.5 and 5 mg/kg/day for 28 days) significantly prolonged the life span of mice transplanted with MV4-11 leukemia (*p* < 0.05 and 0.01, respectively). The median survivals for the control and 2.5 mg/kg treatment group were 34.5 and 43 days, respectively. The higher dosage treatment caused an increased antitumor efficacy, with the median survivals for the two groups being 45 and 60 days, respectively

Given its very high cellular activity, compound **1** was tested for its in vivo antileukemia activity in a mouse model of MV4-11 leukemia. Previous in vivo pharmacokinetics studies of compound **1** showed that it is degraded rapidly in mice, having a short half-life (~1 h) in the plasma [30]. Continuous infusion of **1** using a subcutaneously implanted osmotic pump was used for in vivo studies to avoid the unfavorable pharmacokinetics because this route may provide a stable plasma drug concentration. Compound **1** at 2.5 and 5 mg/kg/day for 28 days did not cause weight losses as well as any visible signs of toxicity in mice. Choosing 28-day drug administration was because of the slow antiproliferation activity of compound **1**. A blood test on day 28 showed no significant differences in blood cell counts as well as hemoglobin between mice in the treatment and control groups (Fig. 5a).

A mouse model of systemic MV4-11 leukemia was established by injecting 10^7^ MV4-11 cells/mouse intravenously through the tail veil into NOD-SCID mice with an engraftment rate of 100 %. After 2 weeks, human CD33^+^/CD45^+^ leukemia cells (0.1–0.5 %) can be detected by FACS in blood samples of the mice. The disease progressed rapidly, causing deaths in ~6 weeks after leukemia transplantation. Thus, upon detection of MV4-11 engraftments, mice were randomly separated into treatment and control groups. Mice (*N* = 5) were treated with **1** using dosages of 2.5 and 5 mg/kg/day for 28 days, after which mice were sacrificed and their bone marrow, spleen, and blood were analyzed for human CD33^+^/CD45^+^ leukemia cells. As shown in Fig. 5b, compound **1** was able to significantly inhibit the progression of the leukemia. The lower dose treatment reduced MV4-11 leukemia cells by 80 % in the bone marrow (averaging 6.7 % in the treatment vs. 33.6 % in control group). Similarly, compound **1** caused 81 and 92 % less leukemia cells in the spleen and blood, respectively. The antitumor effects were more pronounced in the higher dose treatment group, achieving 96, 89, and 99 % inhibition rates in the bone marrow, spleen, and blood, respectively. In addition, large metastatic or invasive tumors in the gastrointestinal tract and ovaries were observed in the control mice, while none were visible in the treatment groups. In another set of antileukemia activity evaluation, survival experiments showed that compound **1** at the 2.5 and 5 mg/kg dosages can significantly prolong the lifespan of the leukemia-bearing mice (*p* < 0.05, Fig. 5c). The median survivals for the control and 2.5 mg/kg treatment group were 34.5 and 43 days, respectively. The higher dosage treatment caused an increased antitumor efficacy, with the median survivals for the two groups being 45 and 60 days, respectively. These results demonstrate that LSD1 inhibitors have significant in vivo antitumor activity and are potentially useful therapeutics for MLL-rearranged leukemia.

### Microarray studies of LSD1 inhibition

Since methylated H3K4 is an important histone biomarker for gene regulation, we performed microarray studies to determine how potent and selective LSD1 inhibitors **1** and **2**, which were found to increase H3K4me2, affect gene expression in MV4-11 leukemia cells. In addition, this profiling could help find the mechanism(s) by which these compounds inhibit proliferation, induce differentiation, and cause apoptosis of the leukemia cells.

**Fig. 6:**
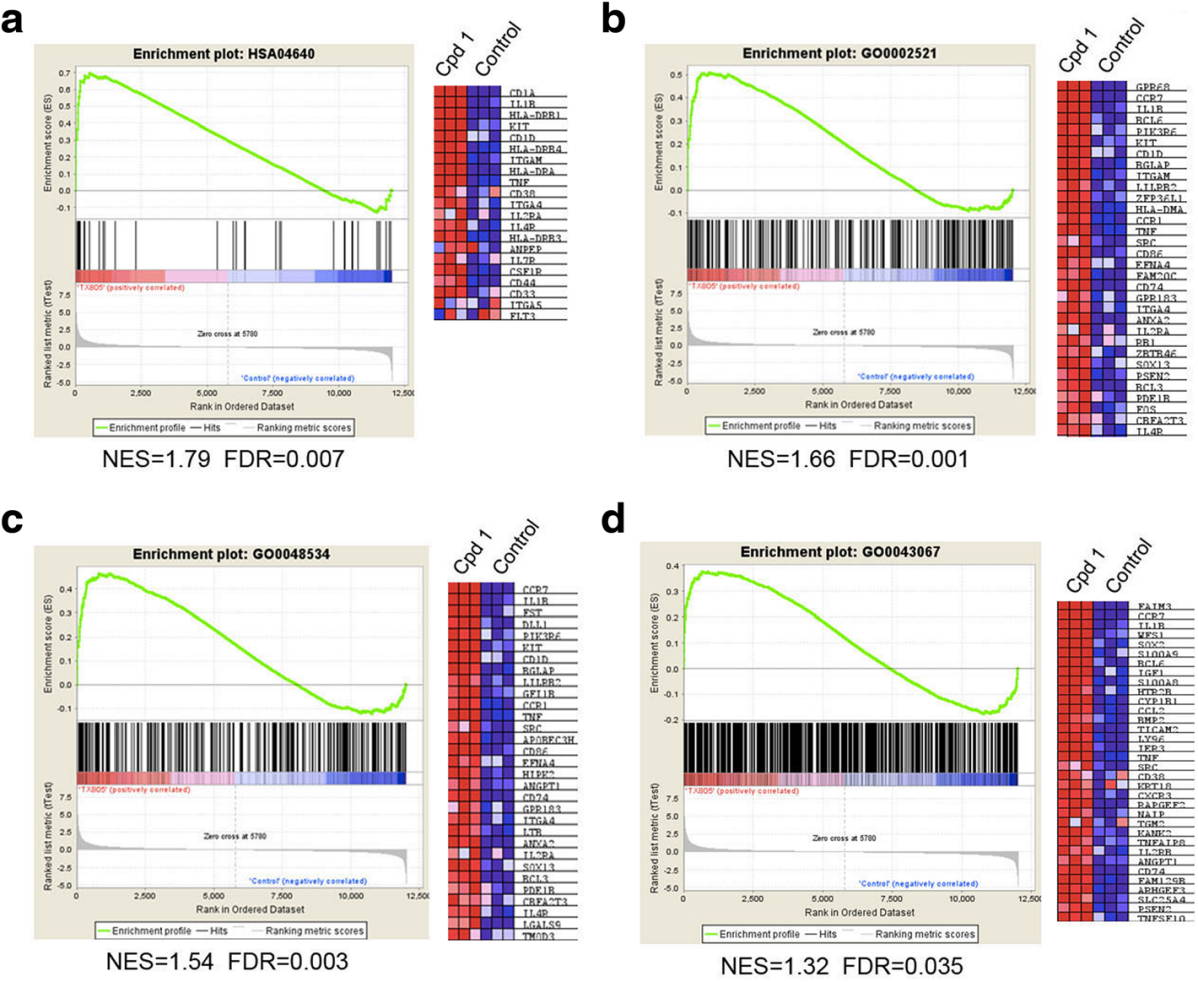
Microarray results of LSD1 inhibition in MV4-11 cells. Upon treatment with compound **1** (100 nM), GSEA plots showed significant upregulation of the gene sets of **a** hematopoietic cell lineage (HSA04640), **b** leukocyte differentiation (GO:0002521), and **c** hematopoietic or lymphoid organ development (GO:0048534), as well as **d** the pro-apoptotic gene set of regulation of programmed cell death (GO:0043067). The *right panels* in **a**–**d** are heat maps showing expression levels of selected genes in the leading edges of the GSEA plots

To this end, triplicate samples of MV4-11 cells were treated with compounds **1** and **2** and the RNA of these samples was isolated, amplified, and hybridized to Illumina HT-12 microarrays. The microarray data were log2-transformed and normalized to have the same median values for comparative analysis. Moderate *t* test was applied to search for genes that were differentially expressed between the control and compound treated samples, using the filter thresholds of *p* values <0.05 and fold changes >4. Compared to the untreated controls, compounds **1** and **2** were found to cause highly similar changes in gene expression pattern (Additional file 1: Figure S6) despite their different chemical structures, suggesting that LSD1, the common target of these two compounds, is responsible for the cellular activities. Next, we used gene set enrichment analysis (GSEA) in an effort to find the possible mechanism(s) for the LSD1 inhibition-mediated antileukemia activity. Expression of several gene sets/pathways related to hematopoietic differentiation as well as apoptosis was found to be significantly affected by LSD1 inhibition. As shown in Fig. 6a–c and Additional file 1: S7A–C, treatment with compounds **1** and **2** resulted in significant upregulation of the gene sets of hematopoietic cell lineage (HSA04640), leukocyte differentiation (GO:0002521), and hematopoietic or lymphoid organ development (GO:0048534), whose expression is important for the differentiation of hematopoietic stem or progenitor cells to become more matured lineages of blood cells as well as the development of hematopoietic organs. These results are consistent with our experimental observations showing that compounds **1** and **2** promoted considerable differentiation of MV4-11 cells. In addition, as shown in Fig. 6d and Additional file 1: S7D, the two LSD1 inhibitors also caused significant upregulation of the pro-apoptotic gene set of regulation of programmed cell death (GO:0043067), which could lead to the observed increased apoptosis of MV4-11 cells upon treatment of these compounds.

## Discussion

MLL translocations are found in ~75 % infant and 10 % children/adult acute leukemia showing a poor prognosis, with 5-year event-free survivals being <40 % [3–5]. The phenotype of MLL-rearranged leukemias can be ALL, AML, or mixed lineage leukemia. However, despite the different phenotypes as well as the variety of fusion partner genes (>70), this subtype of acute leukemias show a similar gene expression profile [42]. Therefore, MLL translocations can serve as a distinct biomarker in the clinic as well as for drug discovery and development [43]. There is a pressing need to find new drugs, especially less toxic therapeutics targeting onco-MLL, which loses the C-terminal SET domain that methylates H3K4. LSD1, which is able to demethylate H3K4me1 and 2, has been found in the MLL transcription complex and counteracts the SET domain of MLL. It is therefore assumed that MLL and LSD1 can keep a balanced methylation levels at H3K4, which is a critical “histone code” for active transcription [7, 8]. In MLL-rearranged leukemia, the balance in H3K4 methylation is impaired. In addition, studies have shown the majority of MLL fusion genes, including AF4, AF9, AF10, and ENL, can recruit DOT1L and methylate H3K79. This aberrant histone methylation at the MLL target gene loci causes overexpression of many Hox genes (e.g., HoxA7, HoxA9, and Meis1) that eventually cause leukemia initiation. More intriguing is that a recent report showed that the H3K4 methylation function of the wild-type MLL in the other allele is essential for MLL-rearranged leukemia [44]. It seems that the complicated “histone codes” at H3K4 and K79 in this malignancy need to be corrected. Pharmacological inhibition of DOT1L has been found to be an effective approach to the treatment of this subtype of leukemia, due to reduced H3K79 methylation levels that inhibit expression of the above leukemia relevant genes. A potent DOT1L inhibitor has been in clinical trials against MLL-rearranged leukemia [20]. More recently, LSD1 has been suggested to be a potential drug target for MLL-rearranged leukemia. However, as described in the “Background” section, more studies using potent and selective LSD1 inhibitors are needed to further validate this hypothesis.

In this work, a series of potent LSD1 inhibitors were synthesized and found to have very potent activity against proliferation of MLL-rearranged leukemia cells MV4-11 and Molm-13 with EC_50_ values of 10–320 nM, while these compounds had no or considerably weakened activity against proliferation of several other leukemia and solid tumor cells. Next, more medicinal chemistry studies showed that antileukemia activity of these compounds correlated with their LSD1 inhibitory activity, suggesting that LSD1 is the cellular target. Excellent LSD1 selectivity (200–>1,000-folds) against related MAO-A and -B enzymes is also a desirable feature of these compounds in the context of drug discovery. Two most potent LSD1 inhibitors **1** and **2** were found to be cell membrane permeable and can significantly increase the levels of H3K4me2 in MV4-11 cells. The most potent compound **1** showed significant antitumor activity in a systemic MV4-11 leukemia mouse model without overt toxicities to mice. Upon treatment (5 mg/kg), the leukemia burdens in the bone marrow, spleen, and peripheral blood were inhibited by >89 % and the life spans for the experimental animals were significantly prolonged, showing the potential of this class of compounds to become clinically useful therapeutics for MLL-rearranged leukemia.

More biological studies demonstrated that LSD1 inhibitors **1** and **2** were able to downregulate the expression of leukemia-relevant genes HoxA9 and Meis1, induce apoptosis, and differentiation. In addition, the ability of these compounds to inhibit colony-forming ability of primary MLL leukemia patient cells suggested that LSD1 inhibition impaired self-renewal of the stem-like leukemia cells. Microarray studies showed that LSD1 inhibition by compounds **1** and **2** caused significant upregulation of several gene sets that promote hematopoietic differentiation and apoptosis, which revealed the underlying mechanisms of LSD1 inhibition in MV4-11 leukemia cells.

Given the promising in vitro and in vivo antitumor activity, compound **1** represents a potential therapeutic agent against MLL-rearranged leukemia. MLL gene rearrangement could serve as a criterion for patient recruitment in a future clinical trial. More preclinical evaluation, especially animal pharmacokinetic studies, should be performed to further evaluate whether **1** as well as other LSD1 inhibitors are viable drug candidates. In addition, MLL translocations can also be found in a small portion (<1 %) of chronical myeloid leukemia (CML) patients [45]. Although rare, these patients had a low response rate to BCR-ABL inhibitor therapies and a poor prognosis. Evaluation of potent inhibitors of LSD1 (or DOT1L) against this molecular subtype of CML might be useful. It is also noted that LSD1 has been found to play a critical role in development [23]. Germline knockout of LSD1 is embryonically lethal. Conditional knockout in adult mice caused blocked hematopoiesis and pancytopenia. In addition to H3K4 and H3K9 [46] as well as other non-histone proteins such as DNMT1 (DNA methyltransferase 1) [47] are substrates of LSD1. For example, LSD1 is of importance to the stability, integrity, and function of DNMT1, an enzyme that maintains appropriate DNA methylation. LSD1 knockdown was found to cause global DNA hypomethylation [47]. These lines of evidence raise a safety concern of LSD1 inhibition. Nonetheless, another conditional LSD1 knockout mouse model study showed that after termination of LSD1 knockout, the impaired hematopoiesis can be fully recovered in the experimental animals [48]. Here, we further demonstrated that potent LSD1 inhibitor **1** can be safely administered (5 mg/kg/day) for an extended period of 28 days without overt toxicities to mice. Significant antitumor activity has also been observed. These experiments strongly suggest that there is a sufficient therapeutic window for pharmacological inhibition of LSD1 targeting leukemia.

Moreover, combination therapy could be a viable approach to further reduce the potential toxicity issue of LSD1 inhibitors. We show in this study that combination treatments of LSD1 inhibitors with a DOT1L inhibitor SYC-522 exhibited strong synergism against proliferation of MLL-rearranged leukemia cells, presumably because H3K79 hypermethylation is closely associated with imbalanced H3K4 methylation in MLL-rearranged leukemia. In addition, since inhibition of LSD1 also caused DNA hypomethylation, the combination of an LSD1 inhibitor with a DNMT inhibitor (e.g., 5-azacitidine or decitabine) could also be effective.

## Conclusions

LSD1 is a drug target for MLL-rearranged leukemia and small molecule LSD1 inhibitors are potential therapeutics for the malignancy.

## Methods

### Cell lines and primary cells

MLL-rearranged leukemia cells MV4-11 and Molm-13 were obtained from ATCC (American Type Culture Collection, Manassas, VA) and DSMZ (Deutsche Sammlung von Mikroorganismen und Zellkulturen GmbH, Braunschweig, Germany), respectively. Non-MLL leukemia cells NB4 and U937 were described in our previous publications [49, 50]. The primary samples were obtained from patients who were treated at Texas Children’s Cancer Center and whose parents consented to the storage of remainder pheresis or bone marrow material for future research, in accordance with the IRB-approved protocol H-3342. The primary leukemia sample is the remainder bone marrow from a child diagnosed with AML with t(11;19).

### Compound synthesis and characterization

All chemicals for synthesis were purchased from Alfa Aesar (Ward Hill, MA) or Aldrich (Milwaukee, WI). The compound identity was characterized by ^1^H NMR on a Varian (Palo Alto, CA) 400-MR spectrometer. The purities of synthesized compounds were determined by a Shimadzu Prominence HPLC with a Zorbax C18 (or C8) column (4.6 × 250 mm) monitored by UV at 254 nm. The purities of the reported compounds were found to be >95 %. Synthesis and characterization of compounds **1**–**4** can be found in Additional file 1.

### LSD1 enzyme inhibition

Human LSD1 catalytic domain (172-833) was cloned and inserted into pGEX-KG vector. The correctness of insert was verified by sequencing. BL21-CodonPlus strain (Agilent) was transformed with the pGEX-KG-LSD1 plasmid and cultured at 37 °C in LB medium containing ampicillin (50 μg/mL) and chloramphenicol (34 μg/mL). Upon reaching an optical density of ~0.9 at 600 nm, LSD1 expression was induced by adding 0.2 mM isopropylthiogalactoside at 25 °C for 20 h. Cells were harvested, lysed, and centrifuged at 20,000 rpm for 20 min, and the supernatant was collected and subjected to an affinity column chromatography using the glutathione sepharose resin. The GST-LSD1 fusion protein was eluted with 10 mM of glutathione solution and purified by chromatography with a Superdex 200 gel filtration column with ~90 % purity (SDS-PAGE).

The inhibition assay for the recombinant LSD1 was performed using a published protocol [30], with the reaction rate (i.e., amount of the product H_2_O_2_) being quantitatively determined by adding HRP and a HRP fluorescence substrate Amplex red. In a 96-well microplate, an increasing concentration (1 nm–100 μM) of an inhibitor was incubated with 30 nM LSD1 in 50 mM phosphate buffer (pH = 7.0) containing 0.01 % Brij-35 for 30 min at 25 °C, before initiation of the reaction by adding 10 μM of dimethylated peptide substrate ARTK(Me_2_)QTARKSTGGKAPRKQKA (K_m_ ~ 10 μM). The total volume of the reaction mixture is 60 μL. After 20 min, 60-μL solution containing HRP (0.01 unit) and Amplex red (80 μM) was added and the fluorescence of each well was determined using a Beckman DTX-880 microplate reader (excitation at 535 and emission at 595 nm). Data were imported into Prism 5.0 (GraphPad), and the IC_50_ values were calculated by using the sigmoidal dose response curve fitting in the software. The reported IC_50_s were the mean values of at least three independent experiments.

### Inhibition of MAO-A/-B

Recombinant human MAO-A and -B were purchased from Sigma. The inhibitory activity was determined using MAO-Glo assay kit (Promega). In brief, following the manufacturer’s protocol, assays were performed in 384-well white plates (Corning) using MAO-A or -B (100 nM) with a final volume of 20 μL. Reactions were quenched after 60 min by adding reconstituted luciferin detection reagent (20 μL/well); 20 min after addition of the detection reagent, the luminescence of each well was measured using Beckman DTX-880 microplate reader. IC_50_ calculation was similar to that of LSD1.

### Antiproliferation assay

Proliferation inhibition assay for suspension leukemia cells was performed using an XTT assay kit (Roche) with our previously described method. Proliferation inhibition assay for solid tumor cell lines MCF-7 and LNCaP was performed using our previous described MTT assay. The antiproliferation EC_50_ values were determined by Prism, and the reported results were the mean values of at least three independent experiments.

### Western blot

With increasing concentrations of a compound for 5 days, 10^6^ cells/well were incubated and histones extracted with the EpiQuik™ Total Histone Extraction Kit (Epigentek) according to the manufacturer’s protocol. Equal amounts of histones (2 μg) were separated on SDS-PAGE and transferred to PVDF membranes. The blots were probed with H3K4-Me2 and H3 primary antibodies (Cell Signaling), followed by antirabbit IgG (Thermo Scientific) secondary antibodies.

### Flow cytometry

For Annexin V apoptosis assay, 10^5^ cells/well were incubated with increasing concentrations of a compound for 7 or 14 days. Apoptosis was determined using the FITC Annexin V Apoptosis Detection Kit I (BD Bioscience) using the manufacturer’s protocol. For other FACS assays, cells were labeled with fluorochrome-conjugated monoclonal antibodies against human CD14, CD11b, CD45, or CD33 (BD Biosciences) according to the manufacturer’s recommendation. Cells were analyzed using a FACS Calibur (BD Biosciences/Applied Biosystems), and data were processed using the program Flowjo (version7.6.5).

### Quantitative real-time PCR

For 3 days, 10^4^ cells/well were incubated with a compound, and the RNA was extracted from cells using RNeasy mini kit (QIAGEN); 100–1000 ng of total RNA was reverse transcribed using iScript™ Reverse Transcription Supermix (Bio-Rad) using the manufacturer’s protocol. Quantitative real-time PCR was carried out using Fast SYBR Green Master Mix (Applied Biosystems) according to the manufacturer’s instructions. Measurements were performed in triplicate, using GAPDH as the reference gene. Real-time PCR was performed using Biosystems Step One Plus detection system. The following sequences of primers are used:

HoxA9 (forward: 5′-AAAAGCGGTGCCCCTATACA-3′; reverse: 5′-CGGTCCCTGGTGAGGTACAT-3′);

Meis1 (forward: 5′-TGGCTGTTCCAGCATCTAACACAC-3′; reverse: 5′-ACTGGTCTATCATGGGCTGCAC-3′);

GAPDH (forward: 5′-GACAAAATGGTGAAGGTCGGTG-3′; reverse: 5′-CTGGAACATGTAGACCATG-3′)

### Colony-forming assay

Human MLL-rearranged AML samples will be obtained from the Children’s Oncology Group (COG) Biopathology Center, in accordance with the IRB-approved protocol H-24170. The colony-forming assay was performed according to our previously published method [40]. After 14 days, the colony number of each culture dish was counted and imported into Prism 5.0 and EC_50_ values were determined by using the sigmoidal dose response curve fitting in the software.

### Cell migration assays

Cell migration assay was performed in a 24-well transwell plate with 8-μm polyethylene terephthalate membrane filters (Falcon cell culture insert; Becton-Dickinson) separating the lower from the upper culture chambers, according to the manufacturer’s instructions. In brief, MV4-11 cells were treated with or without compound **2** (50 nM and 1 μM) for 4 days. Triplicate samples of 10^6^ cells/well were plated in the upper chamber containing serum-free RPMI-1640. RPMI-1640 supplemented with 10 % FBS in the bottom chamber was used to attract cells to move through the filter. Cells were allowed to incubate at 37 °C for 4 h, after which the cells that had migrated through the pores were harvested in the bottom chamber and counted. Results are presented as means ± s.d.

### In vivo activity studies

All of the mouse studies were conducted in strict compliance with the IRB-approved protocol. NOD-SCID mice (4 to 6 weeks old) were obtained from Jackson lab (Bar Harbor, ME, USA) and maintained under specific pathogen-free conditions; 10^7^ MV4-11 cells/mouse were injected intravenously through the tail veil, and animal weights were monitored twice a week. After 2 weeks when the blood samples showing 0.1–0.5 % CD45+/CD33+ human leukemia cells, mice were randomly segregated into control and treatment groups. Mice in the treatment group were implanted subcutaneously in osmotic pumps (Alzet model 2004) containing 1.75 mg of compound **1** in 200 μL of PBS/DMSO (1:1). Animals were sacrificed when >20 % weight loss, hunched posture, ruffled fur, or inactivity was observed. The peripheral blood, spleen, and bone marrow cells were isolated and tested for CD45^+^/CD33^+^ using FACS. Log-rank analysis was used to determine statistical significance of the survival curves using Prism 5.0.

### RNA amplification and microarray data analysis

For 7 days, 10^5^ cells/well were incubated with compounds. Cells were treated with Trizol® Reagent, frozen in liquid nitrogen, and shipped to Asuragen, Inc. (Austin, TX) for microarray experiments. RNA from these samples was isolated, amplified, and hybridized to Illumina Human HT-12 v4 arrays according to the manufacturer’s protocol. Microarray data were log2 transformed and normalized to have the same median for all the arrays. Moderate *t* statistics were used to find genes that were differentially expressed between the samples of interest. Benjamini and Hochberg method was used to correct for multiple comparisons. R and Bioconductor packages were applied for all the statistical analyses (see http://cran.us.r-project.org/, http://www.bioconductor.org/). GSEA analysis was performed using GSEA software from Broad Institute (Boston, MA).

## Additional file

Additional file 1:
**Figures S1-S7**, **Table S1** and Compound synthesis and characterization. (PDF 1450 kb)

## Abbreviations

AML: acute myeloid leukemia
ALL: acute lymphocytic leukemia
FACS: fluorescence-activated cell sorting
FAD: flavin adenine dinucleotide
H3K4: histone H3 lysine 4
H3K79: histone H3 lysine 79
LSD1: lysine specific demethylase 1
LSC: leukemia stem cells
MAO: monoamine oxidase
MLL: mixed lineage leukemia
PCM: (piperazin-1-yl)carbonylmethyl
