# Supplementary material for: Pharmacological inhibition of LSD1 for the treatment of MLL-rearranged leukemia
Source: J Hematol Oncol. 2016 Mar 12;9:24. doi: 10.1186/s13045-016-0252-7 (PMC4789278; doi:10.1186/s13045-016-0252-7)
Supplement: Additional file 1: — Figures S1-S7, Table S1 and Compound synthesis and characterization. (PDF 1450 kb) [file 13045_2016_252_MOESM1_ESM.pdf]

**Additional Files**

# Pharmacological Inhibition of LSD1 for the Treatment of MLL-Rearranged Leukemia

Zizhen Feng,[1] Yuan Yao,[1] Chao Zhou,[1] Fengju Chen,[2] Fangrui Wu,[1] Liping Wei,[1] Wei Liu,[3,4] Shuo Dong,[5] Michele Redell,[3,4] Qianxing Mo,[2,5] and Yongcheng Song[1,2,]*

[1]Department of Pharmacology, [2]Dan L. Duncan Cancer Center, [3]Department of Pediatrics, [5]Department of Medicine, Baylor College of Medicine, 1 Baylor Plaza, Houston, TX 77030, USA.

[4]Texas Children's Cancer and Hematology Centers, 1102 Bates Street, Houston, TX 77030, USA.

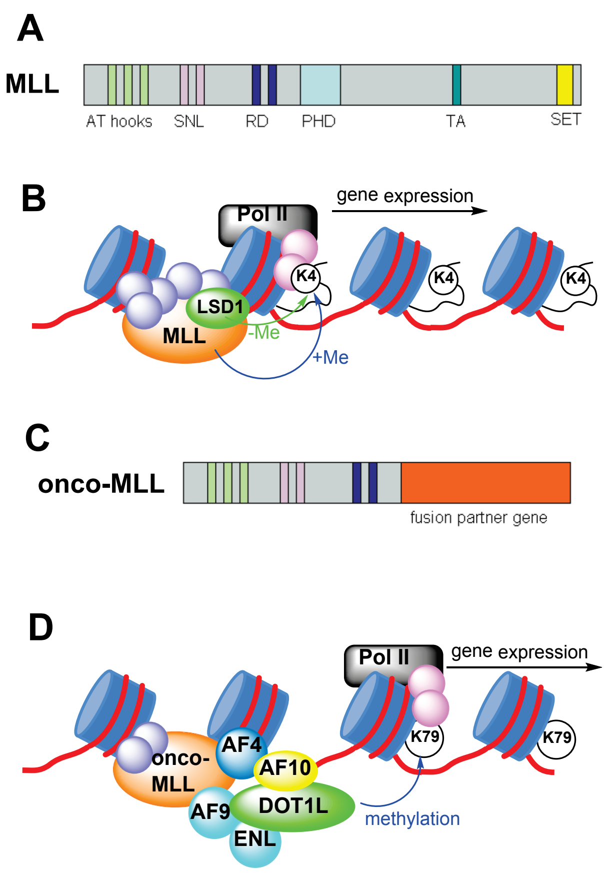


**Figure S1.** (**A**) Illustration of the wild-type MLL protein; (**B**) Functions of wild-type MLL and LSD1. MLL methylates H3K4 and initiates RNA polymerase II (Pol II) mediated gene transcription, while LSD1 removes the methyl group from H3K4-Me1 and 2; (**C**) Illustration of onco-MLL fusion protein; (**D**) Function of onco-MLL protein. The MLL protein complex involving AF4, AF9, AF10 or ENL can recruit DOT1L, which methylates H3K79 and causes overexpression of leukemia-relevant genes.

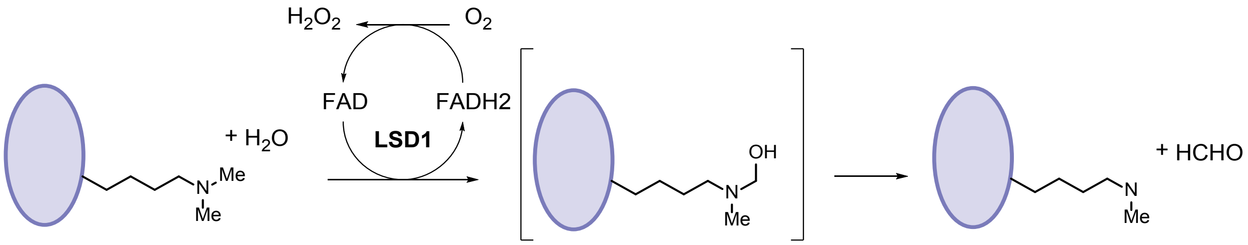


**Figure S2.** Mechanism of catalysis for LSD1.

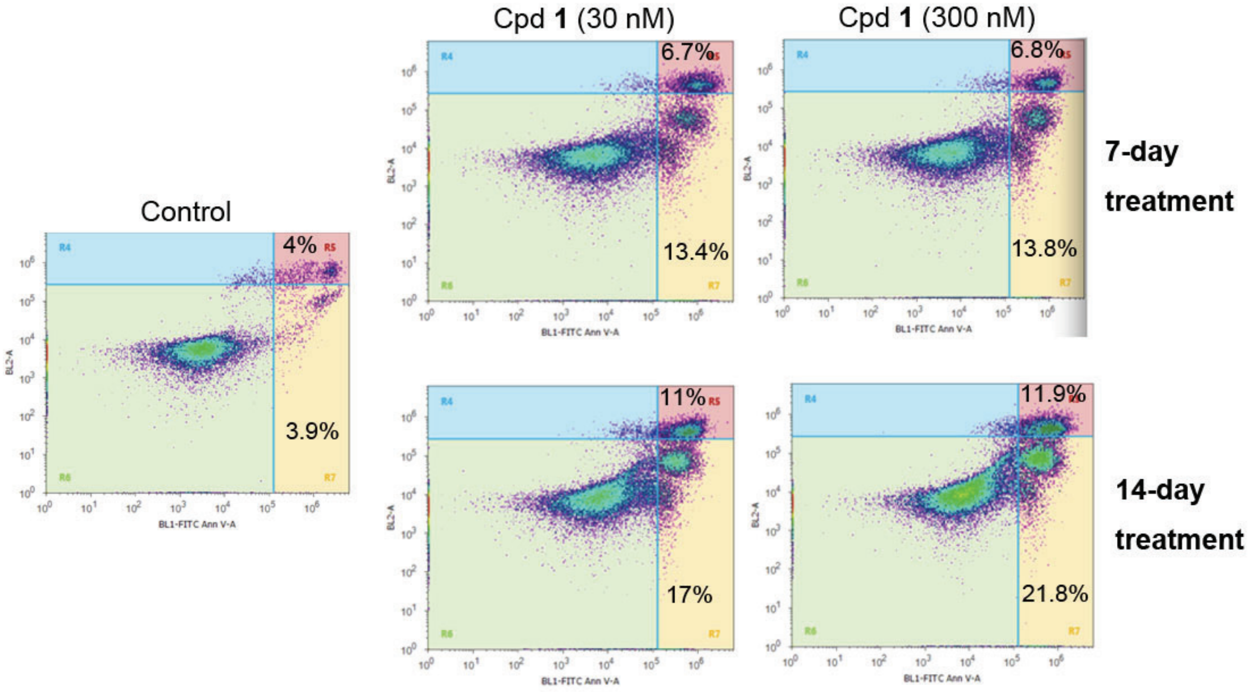


**Figure S3.** After treatment for 7 or 14 days, compound **1** (30 and 300 nM) induced significant apoptosis of MV4-11 leukemia cells. The upper number in each figure refers to the proportion of propidium iodide-positive, apoptosed cells and the lower number refers to that of annexin-V-positive cells (early apoptosis).

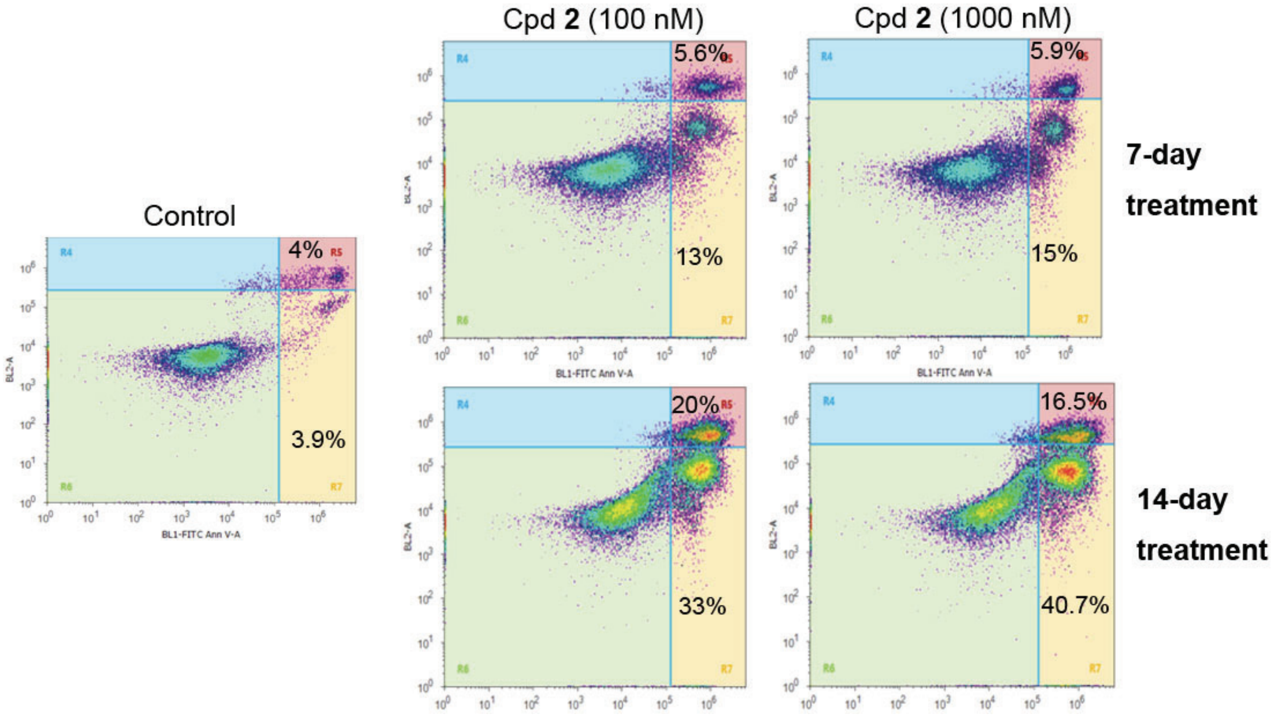


**Figure S4.** After treatment for 7 or 14 days, compound **2** (100 and 1000 nM) induced significant apoptosis of MV4-11 leukemia cells. The upper number in each figure refers to the proportion of propidium iodide-positive, apoptosed cells and the lower number refers to that of annexin-V-positive cells (early apoptosis).

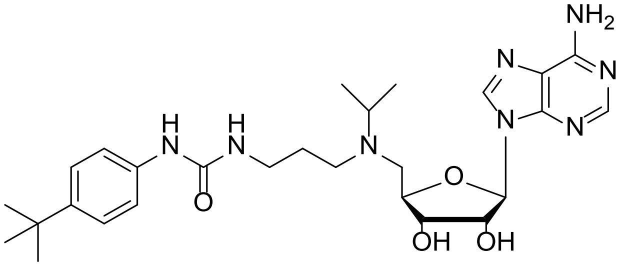


SYC-522

**Figure S5.** Chemical structure of DOT1L inhibitor SYC-522.

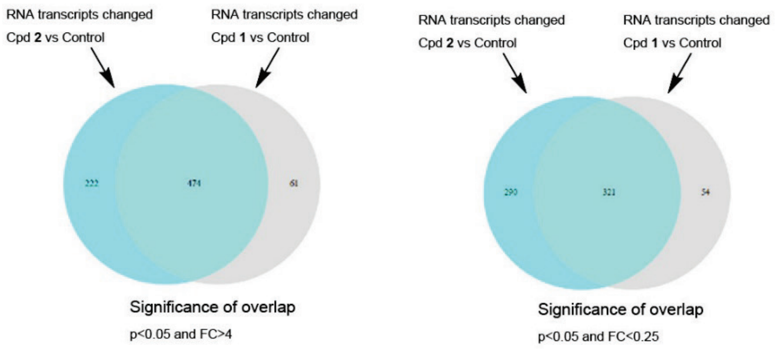


**Figure S6.** Microarray results of LSD1 inhibition in MV4-11 cells show that treatment with compounds **1** and **2** caused highly similar changes in gene expression pattern.

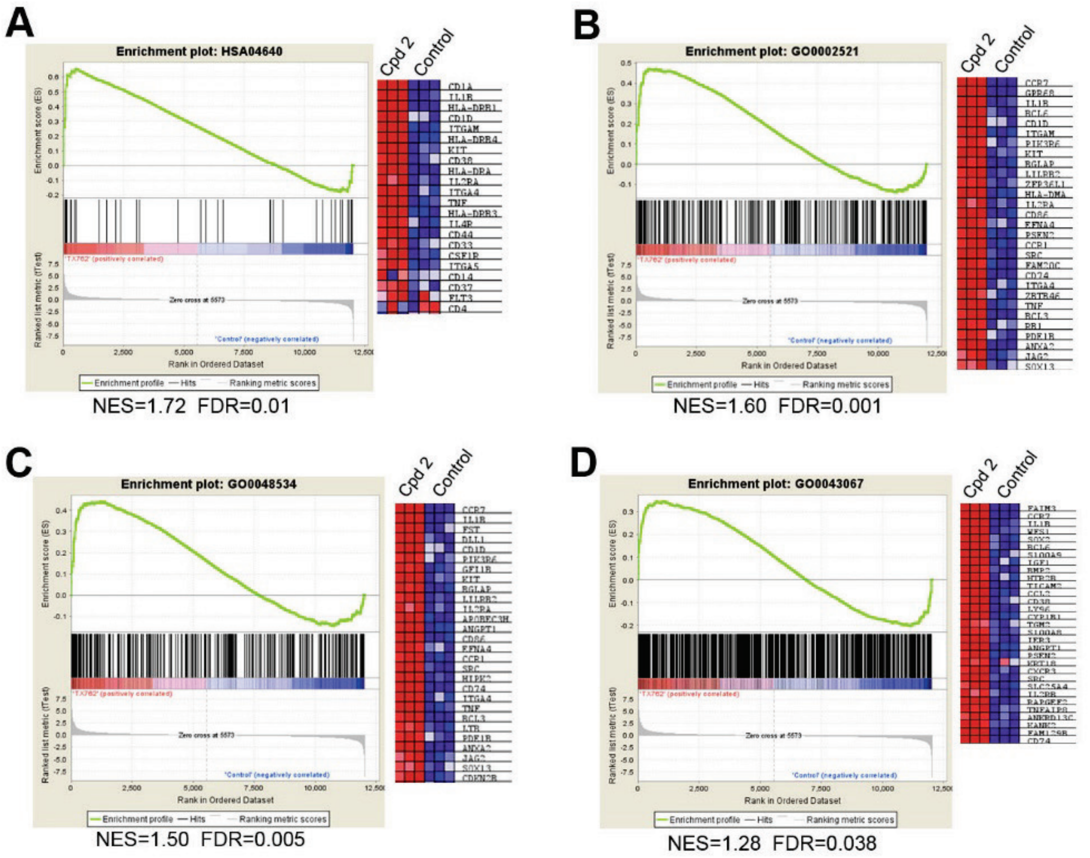


**Fig. S7.** Microarray results of LSD1 inhibition in MV4-11 cells. Upon treatment with compound **2** (300 nM), GSEA plots showed significant upregulation of the gene sets of (**A**) hematopoietic cell lineage (HSA04640), (**B**) leukocyte differentiation (GO:0002521) and (**C**) hematopoietic or lymphoid organ development (GO:0048534), as well as (**D**) the pro-apoptotic gene set of regulation of programmed cell death (GO:0043067). The right panels in (**A**-**D**) are heat maps showing expression levels of selected genes in the leading edges of the GSEA plots.

**Table S1.** Structures and biological activity of compounds **5 - 14**.

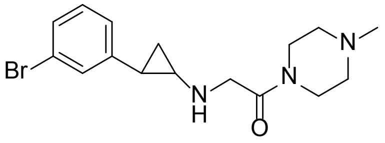

**5**

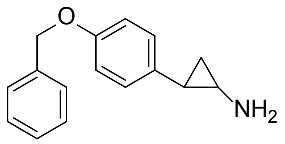

**6**

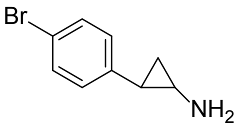

**7**

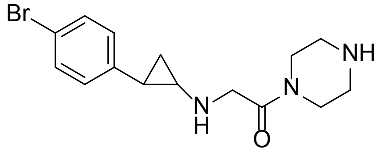

**8**

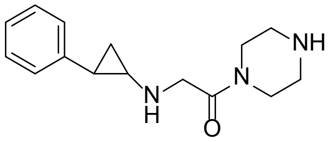

**9**

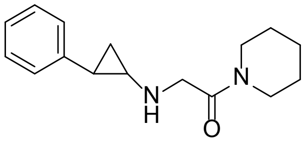

**10**

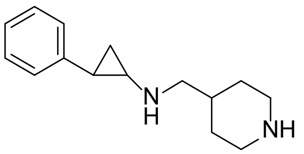

**11**

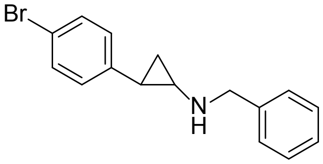

**12**

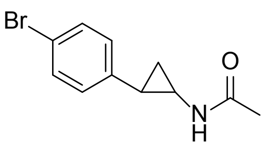

**13**

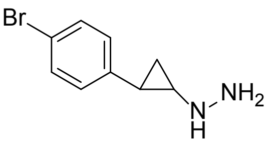

**14**

| Cpd # | LSD1 $IC_{50}$ (μM) | MV4-11 $EC_{50}$ (μM) |
|---|---|---|
| **5** | >100 | 25 |
| **6** | 0.91 | 6.0 |
| **7** | 25 | >50 |
| **8** | 0.064 | 0.10 |
| **9** | 0.19 | 0.28 |
| **10** | 1.3 | 2.5 |
| **11** | 0.062 | 0.13 |
| **12** | 5.0 | 25 |
| **13** | 15.4 | 40 |
| **14** | 5.8 | 42 |

**Compound synthesis and characterization.** All reagents were purchased from Alfa Aesar (Ward Hill, MA) or Aldrich (Milwaukee, WI). Compounds were characterized by $^1$H NMR on a Varian (Palo Alto, CA) 400-MR spectrometer. The purities of all compounds were determined by a Shimadzu Prominence HPLC with a Zorbax C18 or C8 column (4.6 x 250 mm) monitored by UV absorbance at 254 nm, or $^1$H (at 400 MHz) absolute spin-count quantitative NMR analysis using imidazole as an internal standard. The purities of all compounds were found to be >95%.

**Compound 1.** It was prepared from 4-benzyloxy-benzaldehyde and 1-chloroacetyl-4-methylpiperazine, following a published method, as a dihydrochloric acid salt (white powder). $^1$H NMR (400 MHz, DMSO-d$^6$): δ 11.81-11.42 (m, 1H), 9.88-9.54 (m, 1H), 7.49-7.30 (m, 5H), 7.11-6.86 (m, 4H), 5.06 (d, *J* = 10.4 Hz, 2H), 4.48-3.81 (m, 4H), 3.48-3.35 (m, 2H), 3.34-2.86 (m, 4H), 2.74 (s, 3H), 2.59-2.52 (m, 1H), 2.13-2.00 (m, 1H), 1.59-1.50 (m, 1H), 1.21-1.08 (m, 1H).

**Compound 2.** It was prepared from 4-(6-fluoropyridin-3-yl)-benzaldehyde and 1-chloroacetyl-4-methylpiperazine, following a published method, as a dihydrochloric acid salt (white powder). $^1$H NMR (400 MHz, DMSO-d6): δ 8.27 (s, 1H), 8.07 (t, *J* = 8.0 Hz, 1H), 7.53 (d, *J* = 7.2 Hz, 2H), 7.22 (d, *J* = 7.2 Hz, 2H), 7.07 (d, *J* = 8.0 Hz, 1H), 4.63 (d, *J* = 8.4 Hz, 1H), 4.51-4.41 (m, 1H), 4.33-4.22 (m, 1H), 3.93-3.82 (m, 1H), 3.65-3.57 (m, 4H), 3.11-2.98 (m, 4H), 2.83 (s, 3H), 1.55-1.50 (m, 1H), 1.41-1.32 (m, 1H).

**Compound 3.** It was prepared from 4-bromobenzaldehyde and 1-chloroacetyl-4-methylpiperazine, following a published method, as a dihydrochloric acid salt (white powder). $^1$H NMR (400 MHz, DMSO-d6): δ 11.61-11.50 (bs, 1H), 9.88-9.59 (bs, 1H), 7.49 (d, *J* = 8.4 Hz, 2H), 7.16 (d, *J* = 8.4 Hz, 2H), 4.38-4.20 (m, 2H), 3.95-3.81 (m, 1H), 3.66-3.03 (m, 8H), 2.76 (s, 3H), 2.59-2.52 (m, 1H), 1.62-1.57 (m, 1H), 1.29-1.24 (m, 1H).

**Compound 4.** It was prepared from 4-(6-choloropyridin-3-yl)benzaldehyde and 1-chloroacetyl-4-methylpiperazine, following a published method, as a dihydrochloric acid salt (yellow solid). $^1$H NMR (400 MHz, $D_2O$): δ 8.63 (s, 1H), 8.12 (d, *J* = 7.6 Hz, 1H), 7.70 (d, *J* = 7.2 Hz, 2H), 7.61 (d, *J* = 7.2 Hz,

2H), 7.38 (d, $J$ = 7.6 Hz, 1H), 4.71-4.66 (m, 1H), 4.52-4.38 (m, 1H), 4.36-4.18 (m, 1H), 4.07-3.81 (m, 1H), 3.59-3.38 (m, 4H), 3.17-2.93 (m, 4H), 2.84 (s, 3H), 1.55-1.37 (m, 2H).
